# Determinants of symptom relief and quality of life improvement after atrial fibrillation ablation

**DOI:** 10.1093/ehjqcco/qcaf045

**Published:** 2025-07-28

**Authors:** Mark J Mulder, Michiel J B Kemme, Nikki van Pouderoijen, Luuk H G A Hopman, Marthe J Huntelaar, Herbert A Hauer, Giovanni J M Tahapary, Albert C van Rossum, Cornelis P Allaart

**Affiliations:** Department of Cardiology, Amsterdam UMC, Vrije Universiteit Amsterdam, Amsterdam Cardiovascular Sciences, De Boelelaan 1117, 1081HV Amsterdam, The Netherlands; Department of Cardiology, Amsterdam UMC, Vrije Universiteit Amsterdam, Amsterdam Cardiovascular Sciences, De Boelelaan 1117, 1081HV Amsterdam, The Netherlands; Department of Cardiology, Amsterdam UMC, Vrije Universiteit Amsterdam, Amsterdam Cardiovascular Sciences, De Boelelaan 1117, 1081HV Amsterdam, The Netherlands; Department of Cardiology, Amsterdam UMC, Vrije Universiteit Amsterdam, Amsterdam Cardiovascular Sciences, De Boelelaan 1117, 1081HV Amsterdam, The Netherlands; Department of Cardiology, Amsterdam UMC, Vrije Universiteit Amsterdam, Amsterdam Cardiovascular Sciences, De Boelelaan 1117, 1081HV Amsterdam, The Netherlands; Department of Cardiology, Amsterdam UMC, Vrije Universiteit Amsterdam, Amsterdam Cardiovascular Sciences, De Boelelaan 1117, 1081HV Amsterdam, The Netherlands; Cardiology Centers of the Netherlands, 1073TB Amsterdam, The Netherlands; Department of Cardiology, Amsterdam UMC, Vrije Universiteit Amsterdam, Amsterdam Cardiovascular Sciences, De Boelelaan 1117, 1081HV Amsterdam, The Netherlands; Department of Cardiology, North West Clinics, 1815JD Alkmaar, The Netherlands; Department of Cardiology, Amsterdam UMC, Vrije Universiteit Amsterdam, Amsterdam Cardiovascular Sciences, De Boelelaan 1117, 1081HV Amsterdam, The Netherlands; Department of Cardiology, Amsterdam UMC, Vrije Universiteit Amsterdam, Amsterdam Cardiovascular Sciences, De Boelelaan 1117, 1081HV Amsterdam, The Netherlands

**Keywords:** Atrial fibrillation, Catheter ablation, Patient-related outcome measure, Pulmonary vein isolation, Symptoms

## Abstract

**Aims:**

The primary goal of atrial fibrillation (AF) ablation is to improve AF-related symptoms and quality of life. Previous studies have observed a discrepancy between objective recurrence of AF after ablation and patient-perceived change of symptoms. Although predictors of freedom of AF recurrence after AF ablation have been widely studied, factors associated with symptom relief and quality of life improvement remain underexplored. The present study aimed to investigate determinants of symptom reduction and improvement in quality of life after AF ablation.

**Methods and results:**

A total of 382 AF patients (68% paroxysmal AF, 67% male, mean age 63 ± 9 years) undergoing AF ablation were included. Patient-reported outcomes were assessed using the Toronto Atrial Fibrillation Severity Scale (AFSS) and 36-Item Short-Form Health Survey (SF-36) score. Patients completed both the AFSS and SF-36 score pre-ablation, and post-ablation at 4 months and 1 year follow-up. Atrial tachyarrhythmia recurrence was documented in 139 patients (36%) at 1 year follow-up. AF symptom severity, patient-perceived AF burden and quality of life improved from baseline to 1 year follow-up, particularly in patients without atrial tachyarrhythmia recurrence. Greater baseline AFSS-derived symptom severity and patient-perceived AF burden were associated with a greater improvement of AF symptom severity and patient-perceived AF burden after ablation.

**Conclusion:**

This study shows that patients with lower quality of life and greater AF symptom severity and patient-perceived AF burden benefit most from AF ablation, suggesting that more emphasis should be put on the burden of AF symptoms in clinical decision-making.

Key Learning Points What is already known:

- The primary indication for catheter ablation of AF is symptom relief and improvement of quality of life
- A significant discrepancy exists between the objective recurrence of AF and the subjective symptom relief experienced by patients

What this study adds:

- Greater baseline AF severity and higher perceived AF burden are associated with the largest improvements in both symptoms and quality of life after ablation
- Greater emphasis should be put on the subjective burden of AF symptoms, as it plays a critical role in predicting the benefits of ablation for patients

## Introduction

Atrial fibrillation (AF) is the most common sustained arrhythmia in adults and is associated with disabling symptoms and impairment of quality of life.^1^ Catheter ablation of AF has emerged as an important treatment option in symptomatic AF patients.^2^ Although catheter ablation of AF may reduce hospitalisations and even mortality in selected AF patients with concomitant reduced left ventricular systolic function, the primary indication for catheter ablation is currently symptom relief and improvement of quality of life.^3–5^ However, not all patients undergoing catheter ablation for AF experience symptom relief and improvement of quality of life post-ablation.^3,6–8^ Previous studies have observed a discrepancy between objective recurrence of AF after ablation and patient-perceived change of symptoms and quality of life. Several patients report improvement of symptoms and quality of life despite recurrent AF, whereas others without documented AF recurrence experience no improvement.^3,6,9^ Nevertheless, most studies exploring predictors of AF ablation success have used recurrence of atrial tachyarrhythmias as primary outcome measure,^10^ whereas few have studied predictors of symptom relief and quality of life improvement.^3,7,11–13^ Greater understanding of factors that predict whether patients will experience symptom relief or improvement in quality of life may improve patient selection for AF ablation. The present study aimed to investigate factors associated with symptom reduction and improvement in quality of life after AF ablation.

## Methods

### Patient population

Since December 2017, all patients undergoing index or repeat AF ablation at the VU university medical centre that provide informed consent are included in a prospective registry approved by the local ethical committee. Patient-reported outcomes are assessed in these patients using the Toronto Atrial Fibrillation Severity Scale (AFSS) and 36-Item Short-Form Health Survey (SF-36) score. Individual patients included in the present analysis were recruited between December 2017 and June 2020. Patients lost to follow-up before 1 year from the ablation procedure and patients with insufficient knowledge of Dutch language to fill out the study questionnaires independently were excluded. The present study conforms to the principles outlined in the Declaration of Helsinki.

### Ablation procedures

All ablation procedures were performed under either general anaesthesia, deep sedation, or conscious sedation. The atrial septum was punctured after administration of a heparin bolus to achieve an activated clotting time of 300–400 s. A contact force-sensing ablation catheter (SmartTouch, Biosense Webster) and a circular mapping catheter (Lasso®, Biosense Webster, Diamond Bar, CA, USA) were placed in the left atrium. In patients undergoing first ablation procedure, radiofrequency (RF) lesions were created using either a point-by-point technique or a ‘dragging’ technique in a wide-area circumferential ablation pattern around the ipsilateral pulmonary veins.^14^ Pulmonary vein isolation (PVI) after circumferential ablation was confirmed by demonstrating entry and exit block using the circular mapping catheter. Touch-up RF lesions were created if needed to achieve PVI and PVI was re-assessed after a waiting period of 30 min. Additional left atrial (LA) linear ablation or ablation of complex fractionated atrial electrograms was not performed during index procedures. Ablation of the cavotricuspid isthmus was performed in patients with documented typical atrial flutter. In patients undergoing a redo ablation procedure, each pulmonary vein (PV) was assessed for late electrical reconnection and all reconnected PVs were re-isolated through focal ablation of conduction gaps. LA linear ablation or ablation of complex fractionated atrial electrograms was only performed in patients with atypical atrial flutters, if all PVs showed to be isolated, or in patients with extensive LA scar, as determined using voltage mapping. A contact force of 10–20 g was targeted during ablation. Ablation power was set at 30W for the posterior/inferior wall and at 40W for anterior and roof segments. An automated lesion tagging system (VisiTag™, Biosense Webster) was used to display ablation lesions during ablation procedures.

### Follow-up

All patients were followed in the outpatient clinic for at least 1 year following PVI. ECGs were recorded at 1, 3, 6, and 12 months follow-up and additional ECG recordings or 24–48 h Holter-monitoring were obtained in patients experiencing symptoms suggestive of recurrent tachyarrhythmias. Antiarrhythmic drugs were typically discontinued after 3 months post-ablation. Any documented episode of AF, atrial flutter, or atrial tachycardia after a 90-day blanking period lasting >30 s, or confirmed by a standard 12-lead ECG, was classified as AF recurrence.

Patients were asked to fill out both the AFSS and SF-36 pre-ablation, and post-ablation at 4 months and 1 year follow-up.

### Outcomes assessment

Baseline patient characteristics, medical history, imaging characteristics, and follow-up data were noted for all patients in an online dedicated data management system (Castor EDC, Castor, Amsterdam, the Netherlands). AF was classified as either paroxysmal or non-paroxysmal according to the latest guidelines.^1^

The AFSS is a validated AF-specific questionnaire that was used in the present study to quantify AF symptom severity, patient-perceived AF burden, and global well-being. The symptom severity score is derived from a 7-question symptom checklist. Symptoms are scored on a five-point Likert scale, resulting in a total AFSS severity score that ranges from 0 to 35 with higher scores indicating worse symptoms. The AF burden score was calculated from four questions about AF episode frequency, duration, and severity resulting in possible scores of 3–30 with 30 indicating higher patient-perceived AF burden. Global well-being was assessed using a single analogue scale ranging from 1 to 10 with 10 indicating the best possible well-being.

The SF-36 is a widely used generic health status questionnaire that measures eight dimensions of health using 36 items ranging from 0 (worst possible health) to 100 (best possible health). Based on these eight dimensions, a physical component summary (PCS) and mental component summary (MCS) were calculated, standardized to a norm with a mean of 50 and a standard deviation of 10.

### Statistical analysis

Continuous variables are presented as mean ± standard deviation in case of a normal distribution; non-normal continuous variables are presented as median [interquartile range]. Categorical variables are expressed as frequency (percentage). Categorical variables were compared using the chi-square test and changes in patient-reported outcome measures during follow-up were compared between groups using a linear mixed model to account for within-patient correlations. Statistical analyses were performed using SPSS (version 26, IBM Corporation, Armonk, NY, USA). *P* values < 0.05 were considered statistically significant.

Univariate and multivariate Cox regression analyses were performed to assess variables associated with AF recurrence during follow-up. Univariate and multivariate linear regression analyses were carried out to assess factors associated with improvement of AFSS-derived symptom severity and AF burden and SF-36 PCS and MCS. For the analysis of improvement in patient-reported outcome measures, linear regression analyses were performed with the difference between the AFSS and SF-36 scores at 1-year follow-up and baseline as the dependent variable. Independent variables included in the linear regression analyses were age, sex, body mass index, index vs. redo procedure, LA dilatation (LA volume index > 34 mL/m²), AF duration, type of AF (paroxysmal/persistent AF), renal dysfunction, congestive heart failure, hypertension, diabetes mellitus, coronary artery disease, valvular disease and the baseline result of the tested score.

## Results

A total of 451 symptomatic patients underwent AF ablation between December 2017 and June 2020 at our institution. Twenty-six patients were excluded due to missing consent or baseline questionnaires, and three due to missing clinical follow-up data. Of the remaining 422 patients, 382 patients (91%) completed at least one of the study questionnaires during follow-up and were included in the present analysis. Eighty-eight patients (23%) underwent a redo procedure, and 294 patients (77%) an index procedure. The mean age was 63 ± 9 years, 255 (67%) patients were male and the mean BMI was 26.8 ± 4.1 kg/m^2^. Paroxysmal AF was present in 259 patients (68%) and ablation was performed after a median of 44 [18–97] months after AF diagnosis. Baseline characteristics were similar between excluded patients and patients included in the final analysis. Detailed baseline characteristics are depicted in *Table 1*.

**Table 1:** Baseline characteristics

Characteristic	Overall (*n* = 382)
Age (years)	63 ± 9
Male	255 (67%)
Body length (cm)	180 ± 10
Weight (kg)	87 ± 15
Body mass index (kg/m²)	26.8 ± 4.1
Redo procedure	88 (23%)
Paroxysmal AF	259 (68%)
AF duration (months)	44 [18–97]
Number of failed AAD	1 [1–2]
Dilated LA	217 (57%)
eGFR < 60 mL/min/1.73 m²	41 (11%)
Congestive heart failure	38 (10%)
Hypertension	159 (42%)
Diabetes mellitus	30 (8%)
Coronary artery disease	46 (12%)
Valvular disease	12 (3%)
History of stroke/TIA	33 (9%)
CHA2DS2-VASC score	1 [1–3]

AAD, antiarrhythmic drugs; AF, atrial fibrillation; eGFR, estimated glomerular filtration; LA, left atrium; TIA, transient ischaemic attack.

### Clinical follow-up

Three hundred eighty patients (99.5%) were alive at 12 months follow-up; two patients died from a non-cardiac cause. Atrial tachyarrhythmia recurrence was documented in 139 patients (36%) after a median of 150 [107–229] days. Recurrence rates were not significantly different between index and redo procedures (35% vs. 41%, *P* = 0.315), but were higher in patients with persistent compared with paroxysmal AF (44% vs. 33%, *P* = 0.027).

Persistent AF and longer AF duration were significantly associated with atrial tachyarrhythmia recurrence in both univariate and multivariate Cox regression analyses (*Table 2*).

**Table 2:** Factors associated with atrial tachyarrhythmia recurrence during follow-up

	Univariate cox regression		Multivariate cox regression	
	Hazard ratio (95% CI)	*P* value	Hazard ratio (95% CI)	*P* value
Age (years)	1.013 (0.993–1.034)	0.191		
Female sex	1.250 (0.884–1.768)	0.206		
Body mass index	0.994 (0.955–1.036)	0.785		
Redo procedure	1.148 (0.785–1.678)	0.477		
Dilated LA	1.287 (0.914–1.812)	0.149		
AF duration (months)	1.004 (1.002–1.006)	**0**.**001**	1.004 (1.002–1.006)	**0**.**001**
Persistent AF	1.468 (1.045–2.060)	**0**.**027**	1.460 (1.040–2.049)	**0**.**029**
eGFR < 60 mL/min	0.647 (0.349–1.199)	0.166		
Congestive heart failure	1.491 (0.919–2.420)	0.106		
Hypertension	1.187 (0.850–1.657)	0.316		
Diabetes mellitus	0.589 (0.280–1.280)	0.186		
Coronary artery disease	0.901 (0.535–1.518)	0.696		
Valvular disease	1.144 (0.468–2.794)	0.768		

Significant *P* values are indicated in bold. AF, atrial fibrillation; eGFR, estimated glomerular filtration; LA, left atrium.

### AFSS patient-reported outcome measures

**Figure 1:**
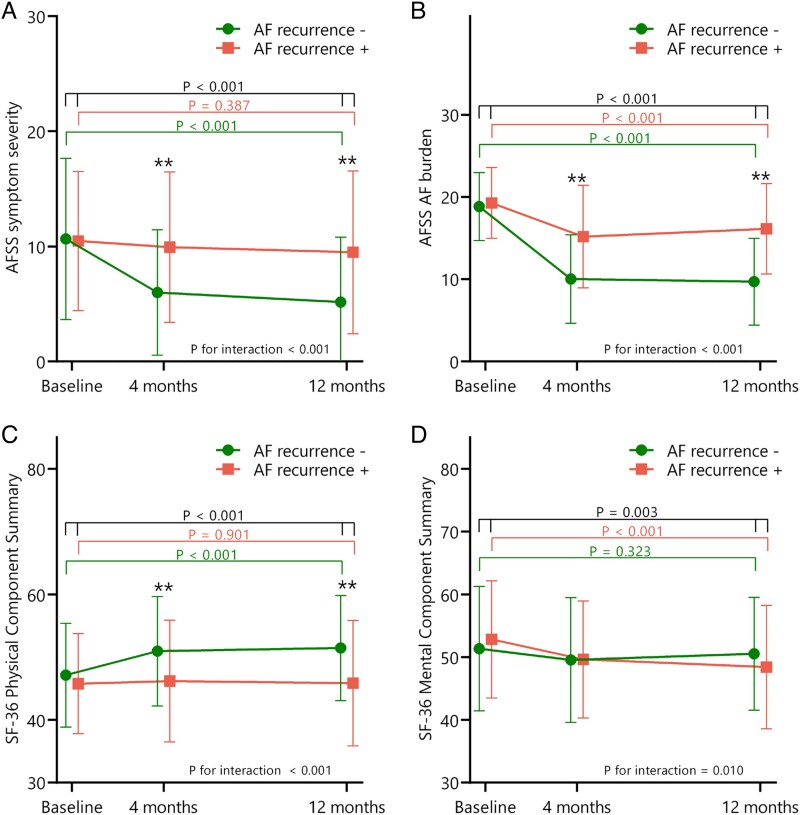
Quality-of-life subscores by recurrence of atrial tachyarrhythmias. Toronto AFSS and SF-36 quality of life scores at baseline, and 4 and 12 months of follow-up, divided by AF recurrence. Changes from baseline to 12 months follow-up are noted with *P* values separately for patients with AF recurrence, without AF recurrence, and all patients combined. AI = Ablation Index. (*A*): Mean values of AFSS symptom severity score (lower = better, range 0–35). (*B*): Mean values of AFSS AF burden score (lower = better, range 3–30). (*C*): Mean values of SF-36 PCS (higher = better, range 0–100). (*D*): Mean values of SF-36 MCS (higher = better, range 0–100). * = *P* value below 0.05, ** = *P* value below 0.01.

Mean AFSS-derived symptom severity, AF burden, and global well-being improved between baseline and 1 year follow-up. Symptom severity score decreased from 10.4 ± 6.5 to 6.6 ± 6.5 (*P* < 0.001), AF burden score decreased from 19.0 ± 4.3 to 11.8 ± 6.2 (*P* < 0.001), and global well-being score increased from 7.2 ± 1.5 to 7.7 ± 1.4 (*P* < 0.001). Thirteen patients (3%) had a baseline symptom severity score of 0. As shown in *Figure 1A* and *B*, baseline AFSS symptom severity and AFSS AF burden did not differ between patients with and without observed AF recurrence during follow-up. At 4 months and 1 year follow-up, both AFSS symptom severity and AFSS AF burden were lower in patients without AF recurrence. Between baseline and 1 year follow-up, patients without AF recurrence demonstrated greater improvements in symptom severity (5.3 ± 6.8 vs. 0.6 ± 7.1; *P* < 0.001) and AF burden (9.1 ± 6.8 vs. 3.2 ± 7.2; *P* < 0.001) compared with patients with recurrent AF. Symptom severity and burden improved in 68% and 61% of patients with recurrence, vs. 90% and 79% in those without.

**Figure 2:**
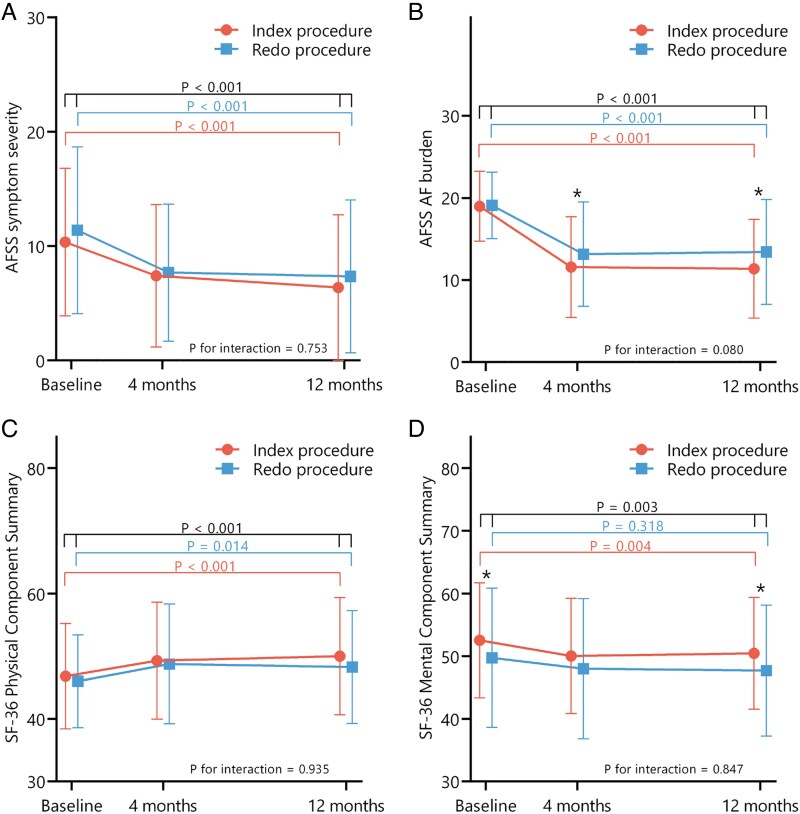
Quality-of-life subscores by index vs. redo procedure. Toronto AFSS and SF-36 quality of life scores at baseline, and 4 and 12 months of follow-up, divided by index procedure vs. redo procedure. Changes from baseline to 12 months follow-up are noted with *P* values separately for patients undergoing index procedure, redo procedure, and all patients combined. AI, Ablation Index. (*A*): Mean values of AFSS symptom severity score (lower = better, range 0–35). (*B*): Mean values of AFSS AF burden score (lower = better, range 3–30). (*C*): Mean values of SF-36 PCS (higher = better, range 0–100). (*D*): Mean values of SF-36 MCS (higher = better, range 0–100). * = *P* value below 0.05, ** = *P* value below 0.01.

Baseline AFSS scores were comparable between patients undergoing index and redo ablation procedures. Both patients undergoing index and redo ablation showed improvements in AFSS symptom severity and AFSS AF burden (*Figure 2A* and *B*). At baseline, females reported worse symptom severity (11.9 ± 6.6 vs. 9.9 ± 6.6, *P* = 0.006) and global well-being (7.3 ± 1.4 vs. 7.8 ± 1.3, *P* = 0.007) than males, while AF burden was similar (19.4 ± 3.7 vs. 18.8 ± 4.4, *P* = 0.183). Improvements in AFSS symptom severity, AF burden, and global well-being were similar in both sexes.

**Figure 3:**
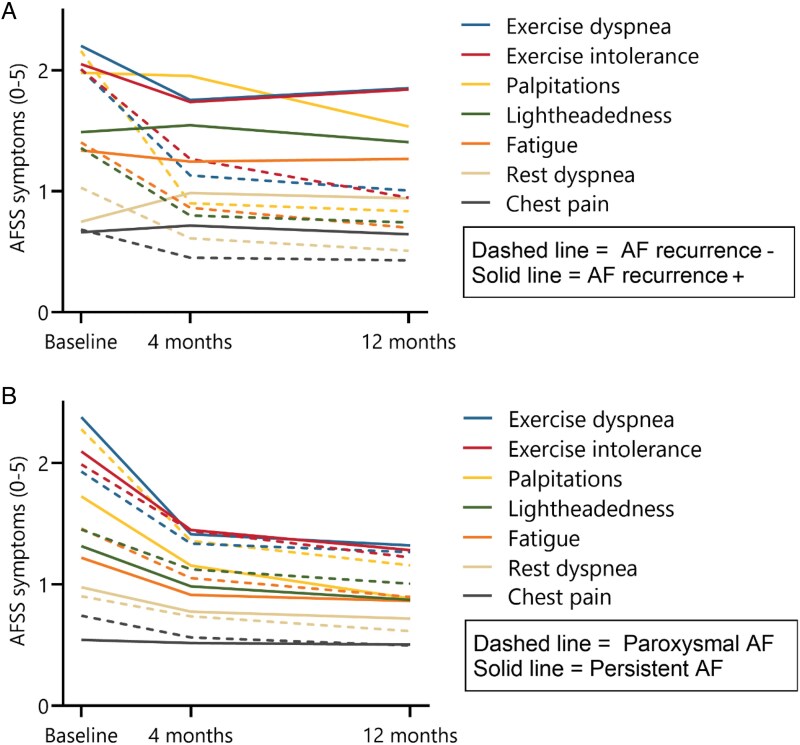
Change in atrial fibrillation-related symptoms over 12 months. Reported atrial fibrillation-related symptoms in part C of the Toronto AFSS at baseline, and 4 and 12 months of follow-up. Lower = better for all symptoms (range 0–5). (*A*): patients divided by recurrence of atrial tachyarrhythmias. (*B*): patients divided by type of atrial fibrillation.

Significant improvements were observed in each of seven individual symptoms assessed in the AFSS questionnaire over 12 months (*Figure 3*).

### SF-36 patient-reported outcome measures

SF-36 physical component score (PCS) improved from 46.7 ± 8.4 to 49.6 ± 9.3 (*P* < 0.001), while the mental component score (MCS) declined from 51.7 ± 10.0 to 49.8 ± 9.3 (*P* = 0.003). At baseline, PCS and MCS did not differ between patients with and without AF recurrence. Improvement in PCS was observed only in patients without recurrence, while MCS decline was confined to those with recurrence (*Figure 1C* and *D*). Changes in PCS and MCS did not differ significantly between index and redo procedures (*Figure 2C* and *D*), though MCS was lower in redo patients at both time points. At baseline, females had lower PCS than males (43.8 ± 9.1 vs. 48.0 ± 7.4, *P* < 0.001), but similar MCS (51.3 ± 9.6 vs. 52.2 ± 9.8, *P* = 0.222). Changes over time were comparable between sexes.

### Factors associated with change in patient-reported outcome measures

Univariate and subsequent multivariate linear regression analyses demonstrated that absence of congestive heart failure and higher baseline AFSS symptom severity were significantly related to a greater improvement in AFSS-derived symptom severity (*Table 3*).

**Table 3:** Factors associated with improvement in AFSS subscales

	Univariate linear regression		Multivariate linear regression	
	Beta (standardized)	*P* value	Beta (standardized)	*P* value
**Improvement in AFSS symptom severity**				
Age	0.025	0.667		
Female sex	−0.073	0.200		
Body mass index	−0.016	0.786		
Redo procedure	−0.019	0.740		
Dilated LA	0.087	0.128		
AF duration (months)	0.025	0.669		
Persistent AF	−0.031	0.587		
eGFR < 60 mL/min	−0.038	0.513		
Congestive heart failure	−0.166	**0**.**004**	−0.132	**0**.**005**
Hypertension	−0.008	0.888		
Diabetes mellitus	0.016	0.781		
Coronary artery disease	0.037	0.523		
Valvular disease	0.013	0.815		
Baseline AFSS symptom severity	0.562	**<0**.**001**	0.554	**<0**.**001**
**Improvement in AFSS AF burden**				
Age	−0.035	0.540		
Female sex	−0.094	0.101		
Body mass index	−0.020	0.733		
Redo procedure	−0.118	**0**.**037**	−0.115	**0**.**014**
Dilated LA	0.025	0.668		
AF duration (months)	0.010	0.856		
Persistent AF	0.088	0.124		
eGFR < 60 mL/min	−0.004	0.942		
Congestive heart failure	−0.070	0.223		
Hypertension	−0.070	0.218		
Diabetes mellitus	0.002	0.971		
Coronary artery disease	0.067	0.242		
Valvular disease	−0.070	0.222		
Baseline AFSS AF burden	0.572	**<0**.**001**	0.571	**<0**.**001**

Significant *P* values are indicated in bold. AF, atrial fibrillation; AFSS, Toronto Atrial Fibrillation Severity Scale; eGFR, estimated glomerular filtration; LA, left atrium.

Index procedure and high baseline AFSS-derived AF burden were predictors of greater improvement in AFSS-derived AF burden.

**Figure 4:**
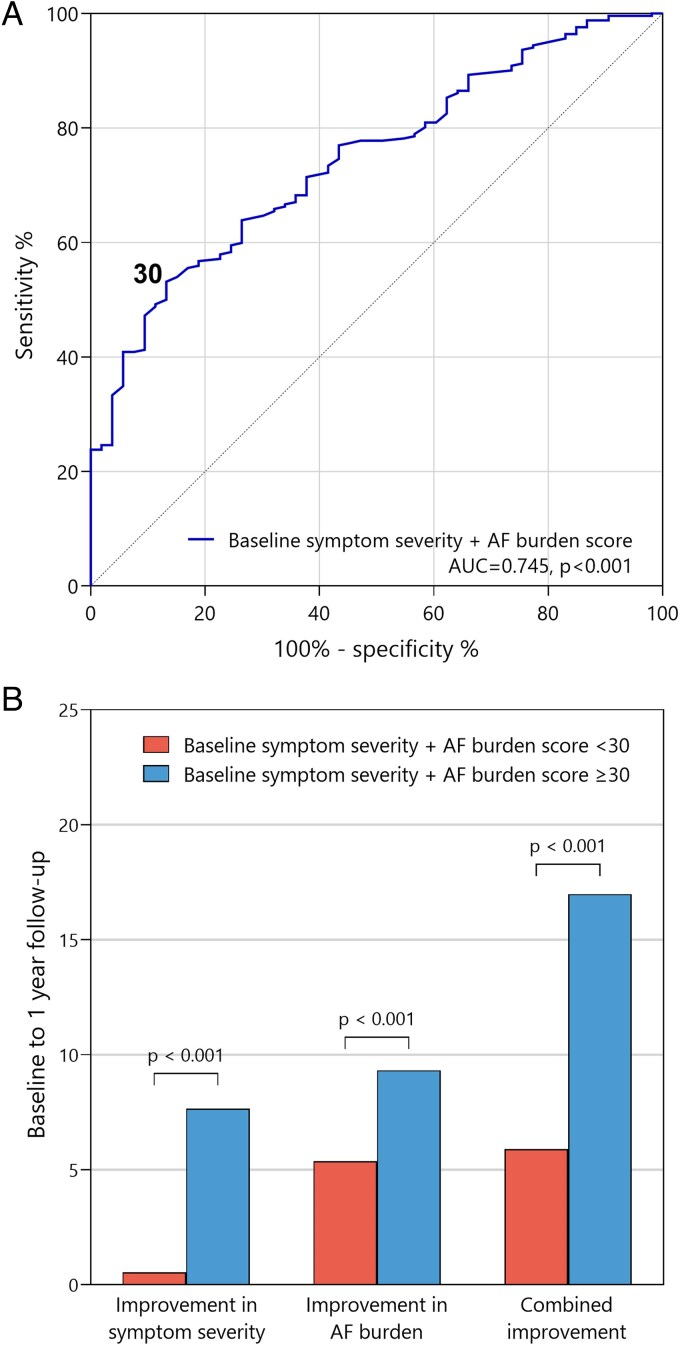
Prediction of AFSS score improvement using baseline scores. Toronto AFSS symptom severity and atrial fibrillation burden scores were added up at baseline and at 1 year follow-up to calculate combined AFSS scores. (*A*): Receiver operating characteristic curve for the prediction of improvement of combined AFSS score between baseline and follow-up using baseline combined AFSS score. (*B*): improvement of AFSS symptom severity, AF burden, and combined AFSS score between baseline and follow-up stratified by baseline combined AFSS score <30 and ≥30. AUC, area under curve.

A combined score of the AFSS subscores (calculated by adding up baseline AFSS symptom severity and AF burden score) demonstrated acceptable predicting ability for improvement of both AFSS scores at 1 year follow-up (*Figure 4A* and *B*). A combined score of 30 was the ideal cut-off as determined by the Youden index.

Regarding SF-36 outcomes, shorter AF duration and lower baseline PCS predicted greater PCS improvement. Absence of heart failure and lower baseline MCS were associated with greater MCS improvement (*Table 4*).

**Table 4:** Factors associated with improvement in SF-36 component summaries

	Univariate linear regression		Multivariate linear regression	
	Beta (standardized)	*P* value	Beta (standardized)	*P* value
**Improvement in SF-36 PCS**				
Age	0.082	0.153		
Female sex	0.069	0.228		
Body mass index	0.034	0.561		
Redo procedure	0.028	0.625		
Dilated LA	−0.010	0.857		
AF duration (months)	0.114	**0**.**048**	0.118	**0**.**026**
Persistent AF	−0.046	0.427		
eGFR < 60 mL/min	−0.035	0.554		
Congestive heart failure	0.035	0.550		
Hypertension	0.045	0.431		
Diabetes mellitus	0.019	0.739		
Coronary artery disease	0.005	0.937		
Valvular disease	0.062	0.286		
Baseline SF-36 PCS	0.385	**<0**.**001**	0.386	**<0**.**001**
**Improvement in SF-36 MCS**				
Age	0.096	0.095		
Female sex	0.042	0.469		
Body mass index	−0.021	0.720		
Redo procedure	−0.016	0.778		
Dilated LA	0.019	0.743		
AF duration (months)	0.026	0.648		
Persistent AF	0.007	0.904		
eGFR < 60 mL/min	−0.037	0.534		
Congestive heart failure	0.108	0.061	0.123	**0**.**007**
Hypertension	0.049	0.393		
Diabetes mellitus	0.016	0.779		
Coronary artery disease	−0.080	0.166		
Valvular disease	−0.047	0.416		
Baseline SF-36 MCS	0.602	**<0**.**001**	0.605	**<0**.**001**

Significant *P* values are indicated in bold. AF, atrial fibrillation; eGFR, estimated glomerular filtration; LA, left atrium; SF-36, 36-Item Short-Form Health Survey.

## Discussion

The present report investigated determinants of symptom reduction and improvement in quality of life after AF ablation. The main findings are as follows: (i) improvement of patient-reported AF symptom severity, AF burden, and physical health were observed at 4 months follow-up and sustained at 1 year follow-up; (ii) greater baseline AF symptom severity and AF burden were associated with a greater improvement of symptom severity and AF burden after ablation; (iii) freedom of documented recurrent AF was associated with a greater improvement of patient-reported AF symptom severity, AF burden, and health; (iv) persistent AF was associated with AF recurrence after ablation, but not with a smaller improvement of AF symptom severity or patient-reported AF burden.

Previous research established that persistent AF and AF duration are risk factors for post-ablation AF recurrence.^15,16^ Accordingly, persistent AF and AF duration were independently associated with recurrent AF in our study. Current guidelines and consensus statements distinguish between paroxysmal and non-paroxysmal or persistent AF for the recommendations for catheter ablation.^1,2^ However, previous studies have focused mainly on the impact of AF type and AF duration on AF recurrence rather than on patient-reported symptoms or quality of life. As shown in *Figure 3B*, both patients with paroxysmal AF and persistent AF demonstrated improved AF symptoms and AF burden after ablation, supporting data obtained in previous studies.^13,17–19^ Although the type of symptoms may differ between patients with paroxysmal and persistent AF, both groups experienced similar improvements. Similarly, AF duration was not associated with change in AF symptoms nor AF burden.

There were no clinical parameters that were associated with improvement of more than one of the studied scores/subscales. In line with findings from Pezawas *et al*.^13^ we found that patients undergoing repeat procedures experienced a similar improvement in AF symptoms compared with patients undergoing index procedure, although the reported improvement in AF burden was smaller in patients undergoing a repeat procedure. The only parameters that were consistently found to be associated with change of all studied scores/subscales, were its respective baseline scores. The inability to predict improvement of AF symptoms and patient-reported AF burden with other parameters than baseline scores is in line with the difficulty to predict other AF ablation outcomes using clinical parameters.^20^ Multiple factors may contribute to the difficulty of predict AF ablation outcomes, including the inability to durably isolate the PVs and not targeting the arrhythmia substrate with current ablation strategies.

Existing research is contradictory regarding the impact of sex on AF ablation outcomes. A meta-analysis including 19 observational studies showed that females undergoing AF ablation had a higher risk of both procedural complications and recurrent AF after ablation.^21^ However, results from the large randomized CABANA trial did not confirm this association, demonstrating similar outcomes on efficacy and safety for males and females.^22^ Similarly contradicting results have been found in studies on sex-related differences in AF symptoms and quality of life after AF ablation. Barmano *et al.*^7^ found that female sex was a significant predictor of a greater improvement of AF symptoms, whereas other studies^23,24^ did not observe differences in the change of symptoms or quality of life between males and females. Our study demonstrated that although females reported worse AF symptom severity, global well-being and psychical health at baseline, sex was not associated with changes of any of the outcomes, including recurrent AF, between baseline and follow-up.

Patient selection for AF ablation remains a major challenge in clinical practice. Most studies have focused on predicting AF recurrence after AF ablation, whereas improvements in symptoms or patient-perceived health may be more meaningful to patients. In this study, the best predicting parameter for symptom and health improvement was its corresponding baseline score. This suggests that more emphasis should be put on the burden of AF symptoms in clinical decision-making. When considering whether a patient should undergo ablation, the focus should primarily be on the severity of symptoms and the patient's perceived burden, rather than on clinical parameters or comorbidities. In our study, combined baseline AFSS symptom severity and AF burden score provided acceptable discrimination between patients with and without improvement of AFSS scores. A combined score of at least 30 was found to be the optimal cut-off. A score of 30 points can be caused by relatively mild symptoms continuously due to persistent AF, or relatively severe symptoms that occur once per month due to short lasting paroxysms of AF.

There has been growing attention in the scientific literature on the benefits of early AF ablation.^25^ However, the present study demonstrates that favourable effects can also be observed with longer AF duration, and AF duration did not predict whether a patient will experience symptom relief. Furthermore, the comparable improvement of AF symptoms, burden and health in patients with paroxysmal and persistent AF questions the distinction made in current guidelines based on AF type. Further studies are required to determine the optimal patient selection strategy for AF ablation resulting in the greatest proportion of patients that experience improvement of symptoms and quality-of-life.

### Limitations

The present study was part of a prospective cohort and was conducted in a single centre, and all patients underwent ablation using RF energy. Results of this study may not be extrapolated to patients in other centres and patients undergoing other ablation techniques.

Second, the patient-reported AF burden could not be linked to real AF burden. Follow-up could have been improved by continuous heart rhythm monitoring using an implantable loop recorder, implantable cardioverter-defibrillator, or pacemaker.

Furthermore, recent EHRA consensus statements recommend an 8-week blanking period for AF recurrence.^2^ However, in accordance with guideline recommendations at the time of data collection, this study applied a 90-day blanking period.

Lastly, the present study used both the AF-specific AFSS questionnaire and the generic SF-36 score, which have been used and validated in a large number of studies.^26^ The AFSS is more sensitive to symptom changes directly related to AF ablation, while the SF-36 offers a broader assessment of overall health status. The combined use of both instruments allowed for a comprehensive evaluation of the impact of AF ablation. Nevertheless, there is currently no consensus on how to measure quality of life and AF symptoms, as evidenced by the use of over 30 different instruments in studies evaluating the impact of AF on these outcomes.^27^

## Conclusions

Significant improvements after AF ablation were observed in patient-reported AF burden, symptom severity, and physical health. The present study found that patients with lower quality of life, greater AF symptom severity, and higher AF burden benefit most from AF ablation, suggesting that more emphasis should be put on the burden of AF symptoms in clinical decision-making.

## Data Availability

The data underlying this article will be shared on reasonable request to the corresponding author.
